# Navigating intellectual property (IP): A comparative analysis of Australian universities’ IP policies

**DOI:** 10.1371/journal.pone.0304647

**Published:** 2024-05-30

**Authors:** Hamid R. Jamali

**Affiliations:** School of Information and Communication Studies, Charles Sturt University, Wagga Wagga, NSW, Australia; Macquarie University, AUSTRALIA

## Abstract

The push towards research commercialisation at universities has highlighted the importance of intellectual property (IP) policies in fostering innovation and guiding and managing research commercialisation activities. This paper undertakes a content analysis of intellectual property policies of all (37) Australian public universities, focusing on policy objectives, definition of IP, ownership of IP created by different creators, and distribution of net commercialisation revenues. It is found that all universities assert ownership over staff-created IP, particularly when related to employment or utilisation of university resources. For students, policies tend to balance their rights with university interests, with nuanced approaches for different types of student participation, but the focus of most policies was on postgraduate students engaging in research activities. While some policies had clear arrangements for IP created by visitors and affiliates and Indigenous cultural and intellectual property (ICIP), about a quarter of policies did not specify arrangements for these groups. Revenue sharing arrangements vary but generally award something between a third to a half of net revenue to creators, to both acknowledge their contribution and incentivise further innovation. Policies included a broad spectrum of objectives, from protecting and commercialising IP to fostering innovation and societal benefit, reflecting varying strategies across the higher education sector. Policies could benefit from further clarity in certain areas such as the rights of students or other creator groups. Research is needed to assess the effectiveness of these policies and their influence on innovation and commercialisation activities.

## Introduction

Intellectual property (IP) policies play a pivotal role in guiding the commercialisation of research, while also establishing the norms and principles that govern the distribution of rights and responsibilities within academic institutions [1]. As universities increasingly engage in research commercialisation, understanding the nuances of these policies becomes crucial. Various countries and universities have different rules about IP ownership, and these change over time especially as there is more push towards commercialisation of research at universities. A historical turning point in this area was the Bayh-Dole Act in the United States. The act granted the ownership of inventions made with federal funding to universities (instead of government) and encouraged patens on federally funded inventions. This significantly eased universities’ ability to claim property rights to discoveries funded by federal funds and it further shaped the IP landscape [2].

Universities in different countries have developed IP policies over the years for effective IP management and knowledge transfer and to facilitate research commercialisation [3]. An old review of IP policies in the top 30 research universities in Canada showed that in most cases, IP ownership remained with the creator, while the university’s share of revenue from commercialisation varied, often being negotiable [4]. In France, the introduction of the Innovation Act ("Loi Allegre") led to increased academic institutions’ claims over IP rights on employees’ inventions, often in collaboration with business companies [5]. Meanwhile, Germany’s 2002 reform, abolishing the "professors’ privilege," resulted in a shift towards university-owned patents [6]. A review of patent policies in six Finnish universities uncovered a shift towards academic capitalism, akin to the mature form observed in the United States [1]. This transformation signified the growing commercialisation of academic research. Geunna and Rossi [7] observed a shift in Europe from inventor ownership to institutional ownership of IP rights, especially post-2000. This transformation was influenced by changes in IP rights regulations, as well as broader institutional, cultural, and organisational shifts.

IP policies play a crucial role in shaping the behaviour of academic inventors and the financial outcomes of research endeavours as a review of IP policies in American universities showed that tight or loose control over faculty members and establishing clear monetary incentives for inventors made a difference in patent productivity [8]. There have been a few studies on university IP policies in Canada. A change from a "university-owns" to an "inventor-owns" IP policy at the University of Toronto substantially increased the number of invention disclosures [9]. Hoye also underscored the idea that the interpretation of IP policy incentives is contingent upon various factors, including academic leadership, group norms, institutional culture, and researchers’ experiences with technology transfer support organisations [9]. A survey of Canadian professors on the impact of IP policy characteristics on faculty members’ commercial behaviours surprisingly showed that the ownership regime of inventions did not significantly influence academic inventors’ behaviour. Instead, control rights and income-sharing schemes emerged as primary drivers of formal and informal commercialisation activities [10]. However, a study of patents and IP policies at 54 Canadian universities concluded that policies where inventors own the IP were the most effective for generating patents within these institutions [11]. Kenney and Patton [12] compared the impact of IP policies on research-derived entrepreneurship and found evidence suggesting that universities with inventor ownership policies may be more efficient in generating spin-offs, both in terms of faculty members and R&D investment. A somewhat similar conclusion was made by Tantiyaswasdikul [13] in Canada. Her data indicated that the outcome of commercialisation was influenced by the policies on intellectual property rights. Despite comparable levels of new invention disclosures and patent value, Canadian universities that adopted institutional IP ownership policies appeared to yield a higher quantity of new licenses and patents. She also found that universities, where the IP ownership was held by the inventors, were more likely to create a greater number of new spin-off companies.

A study by Love [14] in the United States revealed that university patent programs may yield a negative rate of return on investments in high-tech patenting. Such programs could potentially hinder research funding, collaboration, and knowledge dissemination, raising questions about the overall effectiveness of IP policies in fostering innovation. Okamuro and Nishimura [15] explored the impact of IP university policies in Japan, formally introduced in 2003, on biotechnology. They found that policies that enabled equitable revenue and royalty sharing, tailored to partners’ needs, enhanced project performance and fostered firm commitment.

As illustrated above there have been some studies on the impact or effectiveness of policies which indicated that some aspects such as the ownership of IP, the share of revenue that goes to the university, and the control rights that the university has over the IP can all affect the commercialisation of research. Moreover, the impact of university patent and IP policies can vary depending on the context, such as the field of research, the stage of technology development, and the culture of the university. However, few studies in the past have reviewed or compared university IP policies and the first step to evaluate their impact is to analyse and understand policies.

### University IP policies and research commercialisation in Australia

Similar to many other countries, the Bayh-Dole Act of 1980 in the USA inspired and stimulated some similar policies in Australia in the late 1980s [16]. All Australian universities probably had an IP policy by the mid-1990s. This is because in the early 1990s, there were emerging claims by university administration to IP of staff and students [17] and there were calls for clarification and minimum standards of protection for academic IP rights [18]. Moreover, the Australian Research Council (ARC) in the ARC/HEC Advice on Intellectual Property in 1995 recommended that all universities should have an IP policy in place by November 1996 and all universities adopted the recommendation [19]. Having an IP policy that covered certain issues was also a requirement of receiving national research funding and all universities certainly had an IP policy by 2001 [20].

Having an IP policy is not sufficient for successful research commercialisation. While the pressure on Australian universities to exploit IP due to decreasing public funding started at least two decades ago when all universities had IP policy [21], Australia still lags behind in research commercialisation [22–24]. In the last five years, Australia’s rank has been declining in the Global Innovation Index and the number of its innovation outputs is small relative to its level of innovation investment [25]. This is while historically Australia’s expenditure on university R&D is proportionally more than most other advanced economies, particularly when compared with business expenditure on R&D [26]. Some experts suggest that the solution might be to remove barriers and increase incentives for academics to innovate and commercialise [27]. Barriers (e.g., complicated commercialisation procedures) and incentives (e.g., creator’s share of revenue) might be rooted in university policies and procedures. Two old surveys of academics and technology transfer officers in Australia showed that about a third of both groups considered weakness in university IP policy as a major barrier or a barrier to the success of research commercialisation [28,29]. Therefore, effective IP policy is paramount for Australian universities in the context of the existing criticism about Australia’s failure in research commercialisation and innovation [23,24] and the government’s initiative to improve the situation. Examples of initiatives are the Medical Research Commercialisation initiative that provides $450 million over ten years from 2023 to support innovative early-stage health and medical research in Australia. It will help researchers transform ideas into life-saving medicines, devices and treatments to help future patients [30]. Another example is a $2.2 billion investment through the University Research Commercialisation Action Plan which is to strengthen the role of university innovation and industry collaboration in Australia’s economic recovery [31].

However, the only proper review of Australian universities’ IP policies was done a quarter of a century ago by Monotti [32]. She examined the IP policies of 30 Australian universities in terms of revenue distribution and IP ownership and found that universities had different formulas for sharing commercialisation profit, with a common approach that usually divided net profits among the inventor, the faculty, and the university. Originators’ shares in policies ranged from 30% to as high as 60%. More recently, Ross [22] as part of her doctoral thesis examined policies and procedures to find out who was the responsible decision maker for certain commercialisation activities such as identification of IP, investigation of IP viability, and finding industry partners.

### Research aim

While developing an effective IP policy is not enough, it is necessary for successful research commercialisation. Effective IP management not only safeguards the intellectual assets of academic institutions but also plays a crucial role in facilitating the transfer of knowledge and technology from academia to industry. In Australia, there is a need to dissect and understand the nuances of universities’ IP policies. Consequently, this paper aims to analyse Australian universities’ IP policies to find out about some of their key features, similarities and variations. The study specifically looks at what intent Australian public universities pursue in their IP policies, how they define IP, how they approach the ownership of IP created by various groups (e.g., staff and students) and finally how they distribute any net revenue generated through research commercialisation. The study aims to provide insights that could inform future policy enhancements, fostering a more effective and equitable ecosystem for research commercialisation in Australia.

## Methods

In conducting this study, a systematic approach was employed, drawing upon content analysis techniques to systematically evaluate and compare the intellectual property (IP) policies of Australian universities. A similar approach was used to analyse open access policies of Australian universities [33]. Content analysis was chosen as the methodological framework for its capacity to uncover relevant information within the content and facilitate cross-document comparisons [34].

### Policy identification

To find IP policies, the Australian Government’s official list of universities [35] was consulted to identify thirty-seven public universities in Australia. The list of these 37 universities can be found in Table 3 in the findings section. Five private universities—Bond University, Carnegie Mellon University (Australian branch), Torrens University, University of Divinity, and University of Notre Dame—were excluded from the study due to their different nature and administration.

Multiple strategies were employed to identify and retrieve IP policies from the selected universities:

- Policy Libraries: For universities with accessible policy libraries, the policy libraries were explored, utilising their search and browse functions to pinpoint relevant IP policies.
- Web-Based Searches: search was conducted on Google, employing primary keywords such as "intellectual property." Furthermore, complementary terms such as "research commercialisation" were examined to uncover potential policies related to these domains. This dual-pronged approach aimed to ensure all relevant policies could be identified.
- World Intellectual Property Organization (WIPO) Database: the "Database of Intellectual Property Policies from Universities and Research Institutions" provided by the World Intellectual Property Organization (WIPO) was also consulted. This resource offered links to the IP policies of various universities.

### Policy collection and snapshot

All of the thirty-seven Australian public universities studied were found to have IP policies or similar documents. All of the identified documents contained ’intellectual property’ in their titles. However, within this dataset, 33 were explicitly named as policies. Two of these were named “policy and procedure”. One document bore the title ’Intellectual Property’ without further specification of document type, but the document’s content confirmed its status as a policy. Additionally, three documents were designated as ’regulations.’ One university (Federation Uni) did not have a policy but only a procedure, however, the content of the procedure was similar to the policies of other universities and therefore, it was included in the analysis. To ensure a consistent dataset for analysis, all identified policies were collected and saved in November 2023. This particular time point was selected to provide a static snapshot for analysis, as policies may undergo revisions and updates over time.

### Analytical framework

For the systematic analysis of these policies, a hybrid approach encompassing both checklist-based and content analysis was employed. The following key information was extracted from each policy document:

- Date of current policy version
- Date of next scheduled policy review
- Responsible office/policy owner
- Applicability recipients of the policy
- IP definition
- Purpose of policy
- IP ownership guidelines
- Revenue distribution

These categories were developed inductively following a review of a sample of IP policies, ensuring that the analytical framework was aligned with the nuances and intricacies of the policies under examination. This methodological approach was designed to facilitate a robust comparative assessment of IP policies.

### Findings

Although most of the 37 policies were generally up-to-date with median being two and mean being three years old, some policies had not been updated for a significant time with the oldest being nine years old and the newest being updated in 2023. Twenty-six of the policies had a date for the next review and in the case of 10 policies, those dates were in the past, the longest being six years overdue, indicating the slow/delayed process of policy revision. The date of first version of the policy was available for 27 policies and the oldest was in 1983 (UNSW). For two policies this date was in 1996–7, for nine policies the first version was in the 2000s and for the remaining 14 policies the first version was in the 2010s.

In terms of the ownership of policies, this information was available for 35 of them. In the case of most universities, the policy was owned by the vice chancellor leadership team, mostly the research portfolio (DVCR or PVCR). Table 1 shows the terms used in the policies for ‘policy owner’.

**Table 1:** Policy owner.

Position	Count
**Deputy / or Pro Vice-Chancellor (research portfolio)**	25
**Vice-Chancellor**	2
**Deputy Vice-Chancellor (Ethics)**	1
**Deputy Vice-Chancellor (Enterprise)**	1
**Director, Research Innovation and Enterprise**	1
**Director, Research Services**	1
**Executive Director (ED), Office of Research Services / ED, Office of Industry Engagement**	1
**Pro Vice-Chancellor Industry & Commercial**	1
**University Secretary**	1
**Vice-President (Research)**	1
Total	35

### Groups to whom IP policies apply

One important aspect of policies is their scope of applicability. Most policies had a direct statement about this. Four policies did not explicitly specify, however, it was implicitly clear in the documents. Students and staff/employees were the most common groups mentioned (35). See Table 2. Two policies that did not use these terms used broad (perhaps vague) terms of ’all university members’ and ’entire university community’. Policies mentioned other groups such as affiliates, visitors, honorary appointments, titleholders, and contractors. Some defined these terms specifically and some included such groups in their definition of staff.

**Table 2:** Groups mentioned in the policies as people to whom the policy applies.

Groups	Count
**Staff/employees**	35
**Students**	35
**Affiliates**	11
**Visitors**	12
**Honorary appointees**	4
**Adjuncts**	2
**Titleholders**	2
**University members**	2
**Academic status holders**	1
**Contractors**	1
**Officers**	1
**University Community**	1

### Definition of IP

Policy documents usually have a specific section for the definitions of key terms used in the policy to avoid any ambiguity or misinterpretation. Given the core concept in an IP policy is the IP itself, one would expect it to be defined in any such policy. Surprisingly two policies did not explicitly define IP (University of Newcastle and Charles Darwin University), and five universities defined ‘IP rights’ and not IP itself. The analysis of the definitions provided showed a range of similarities and a few notable differences.

*Broad and Inclusive Definitions*: Many policies adopted a broad (e.g., CSU) and inclusive definition of IP. They typically encompassed both registered and unregistered rights. Registered rights like "patents" and "trademarks" were frequently mentioned, with patents appearing in 29 policies and trademarks in 32. These definitions often aligned with international standards, such as those outlined by the World Intellectual Property Organization (WIPO). This indicates a common understanding that IP encompasses a range of protection mechanisms, from formal registrations to more intrinsic copyright protections.

*Detailed Enumerations*: Some policies provided detailed enumerations of IP types (e.g., Deakin), explicitly listing various forms of IP types, such as "inventions" (28 mentions), "designs" (32 mentions), "trade secrets" (23 mentions), and "know-how" (17 mentions), indicating recognition of various forms of intellectual contributions. Categories like "discoveries" (6) and "creations" (3) are less frequently mentioned but their presence in a few policies showed the full spectrum of intellectual endeavours that university policies may protect.

*Distinctions and Clarifications*: A few universities made distinctions between IP and related concepts such as moral rights, providing explanations of what is considered IP and what is not. In fact, the name of the policy for one of the universities (CQU) was the Intellectual Property and Moral Rights Policy. These policies clarified the scope of IP and its relationship to other rights, emphasising the protection of moral rights as closely aligned but distinct from IP. "Rights" was one of the most frequently occurring terms with 33 mentions, highlighting that universities focus on the legal rights associated with IP. The term "legal" however, showed up only in two policies, suggesting that two universities explicitly reference the legal frameworks that underpinned IP definitions and rights.

*Research Data as IP*: There was an acknowledgment that IP may exist in research data (e.g., Monash), especially where intellectual effort has been applied to organise or describe the data or where the data is confidential.

### Purpose of policy

University policies usually have sections that outline their purpose, intent or objectives. For this part of the analysis, text related to either the purpose, intent or objectives of policies was used. An analysis of statements in the 37 policies revealed a multifaceted approach to the management and commercialisation of IP arising from research. The overarching themes identified through these statements highlight the varied priorities and strategies of these institutions.

#### Predominant themes

*IP Ownership and Compliance*: The most common theme, present in the policies of 32 universities, underscores a widespread emphasis on clarifying IP ownership and ensuring regulatory compliance.

*Support for Innovation*: A key priority for 25 universities, this theme reflects the institutions’ dedication to fostering an environment conducive to innovation and the generation of new ideas.

*Student and Staff Development*: Highlighted in 21 policies, this theme indicates a focus on providing development opportunities for the community directly involved in research and IP creation.

*Research and Teaching Integration*: Found in 20 policies, it showcases the institutions’ commitment to linking research excellence with teaching, ensuring that the benefits of research activities enrich the educational experience.

*Commercialisation and Economic Impact*: Emphasised by 18 universities, this theme signals an intent to not only create but also capitalise on IP for economic development and commercial gain.

*Advancement and Transmission of Knowledge*: With an occurrence in 14 policies, this theme demonstrates the universities’ role in disseminating knowledge for the public good.

*Management Principles*: Present in 15 policies, suggesting that universities value the governance of IP management and commercialisation processes.

*Protection of Rights*: Acknowledged in 15 policies, highlighting the importance of safeguarding IP rights within the academic setting.

#### Less common themes

*Community Engagement*: This objective was stated in the policies of 10 universities, pointing towards a goal of engaging with the broader community, possibly to ensure that research benefits have a wider societal reach.

*Transparent Management*: Mentioned in six policies, indicating an awareness of the need for clear and accountable processes in handling commercialisation activities.

*Real-world Impact*: Only two policies explicitly articulated the aim of achieving tangible impacts from their research, suggesting that while important, it may not be a central theme in policy objectives.

*Dispute Resolution*: Similarly found in only two policies, indicating that conflict resolution is acknowledged but not a primary concern within most policy frameworks. This might be because universities might include dispute resolution in their IP procedure (not policy) or have specific procedure document just for dispute resolution (e.g., Monash).

### Ownership of IP created by staff

The policies’ content regarding the ownership of intellectual property (IP) created by staff showed all universities asserted ownership of IP generated by staff during the course of employment as part of their duties. Universities also claimed ownership to IP created by staff in a number of other ways. IP generated using university funding, facilities, resources, or IP derived from sponsored or contract research, or university commissioned projects, was also claimed by the university. Moreover, universities own any IP that is created using existing university-owned IP. While the policies generally supported university ownership over staff outputs, they had scope for exceptions which mostly included literary, scholarly (e.g., book, journal articles) and creative works. Additionally, there may be scope within the agreements for special arrangements to be made with staff members.

The overarching theme was that universities seek to retain ownership of IP to facilitate research sponsorship agreements, collaborations, and their educational mission. However, they also recognise the rights of creators through exceptions and the potential for individualised agreements, balancing institutional interests with those of the staff members.

### Ownership of IP created by students

When it comes to the ownership of IP created by students, there were more variations in policies. Thirty-two policies included a blanket statement indicating that the university in principle does not assert ownership of IP created by students and then go on to list conditions under which the university would assert ownership of student-created IP. Five policies did not have blanket statements, instead only listed situations under which they would assert ownership (e.g., a student is also a staff and created IP during their employment).

While only one policy (ACU) explicitly named undergraduate students to say that the university laid no claim on IP created by them, others incorporated conditions applicable to postgraduates and research students. For example, postgraduate students might need to consent to non-exclusive IP rights for the university (e.g. Swinburn). In cases where a student is also employed by the university, IP ownership typically aligns with staff ownership policies, indicating the role of students (as students or employees) in determining ownership rights. Moreover, policies addressed IP ownership in student-involved projects, particularly externally funded or university research activities which often necessitate IP assignment or licensing. Furthermore, universities may assert ownership over student-created IP under specific agreements, joint creations with staff, third-party agreements, or when existing university-owned IP is used. In the case of the commercialisation of student-generated IP, the student is entitled to a share of revenue. Additionally, students may be required to notify university authorities of valuable IP to mitigate legal, commercial, or reputational risks. Students generally retain the right over their thesis (unless subject to a third-party agreement).

### Ownership of IP created by visitors

Visiting academics are typically hired by one institution but spend time at a second university in a research role. Since they may not be employed by the host university, ownership of IP from their work can be complex. Policies were checked whether they specify ownership in such cases. In 10 policies, visitors were not mentioned at all. The analysis of the information on visitors in the other 27 policies showed some variation in how institutions address the intellectual property rights of visiting creators. The universities that included visitors in their policy can be divided into four groups in terms of their approach to visitors’ IP rights.

- Six universities (e.g., DU) stated that the ownership of IP created by visitors is determined by the agreement they sign before their visitation, and five (e.g., CDU) said the IP rights are determined through case-by-case negotiation.
- Four universities (e.g., ANU) took a presumptive exclusion (non-ownership) approach by stating that the university does not assert ownership of IP created by visitors, and then they go on to add conditions under which they would assert ownership of IP (e.g., when visitors use the university’s funding or facility and resources).
- Eleven universities (e.g., Melbourne) took a presumptive inclusion (ownership) approach and stated that the university owns the IP created by visitors. Some (4) of these universities (e.g., SCU) considered visitors as a member of staff and therefore, the same rule applicable to staff (default university ownership) would apply to them. This ownership usually occurs under certain conditions such as the use of university resources, terms of employment and so on. Given the similarity of the conditions in non-ownership (category B above) and this ownership approach, in practice, the outcome of the two approaches might not be very different.
- One university (VU) stated the IP of the work commissioned by the university (including computer programs, learning and teaching material etc will be owned by the university and visitors will own the copyright of any other work they might do.

### Ownership of IP created by affiliates

Another category of researchers who might engage in IP creation is affiliates. Policies were searched for the words affiliates, adjuncts, honorary appointment/appointees, titleholders, and associates to find out how policies approached the ownership of IP created by this creator group. The approach to affiliates mirrored that of visitors. Seven policies (e.g., CDU) made no mention of affiliates, six (e.g., CSU) included them within the definition of staff, and five (e.g., WSY) grouped them with visitors. Ten policies took a presumptive ownership approach and stated that they assert ownership of IP created by this group, but of course, they had conditions under which such ownership would happen. Two policies took the opposite approach, i.e., presumptive exclusion (non-ownership) stating that they do not assert ownership on IP created by affiliates except in certain conditions such as the use of the university’s resources, or the use of university’s IP, teamwork with the university staff and so on. Four other university policies stated that the terms in the agreements signed by affiliates will govern the ownership of their IP. Finally, one university stated that they would negotiate with affiliates about IP cases, and one policy was not clear in this regard.

### Ownership of Indigenous cultural and intellectual property

Indigenous matters are increasingly important in the Australian context and when it comes to Indigenous Cultural and Intellectual Property (ICIP), it is expected that universities have clear guidelines that respect the rights of First Nations people. Eleven policies had no information about ICIP. One of them (Griffith) mentioned Indigenous people in the context of Benefit Sharing Arrangements. The rest of the policies had a section or a statement concerning Indigenous people, some brief and some in more detail. Analysis of these statements revealed a consistent and respectful approach to managing Indigenous Knowledge and IP. The key trends and principles in these statements were:

*Recognition and respect*: Universities (16) explicitly acknowledged the significance and importance of Indigenous Knowledge, with a commitment to respect and protect it. This was often reflected in statements that emphasised the dynamic and living nature of Indigenous Knowledge.

*Ownership and consent*: There was a clear stance (13) against asserting ownership over IP relating to works predominantly created by Aboriginal or Torres Strait Islander people. Policies stipulated that the use of Indigenous Knowledge must have the prior approval of the appropriate knowledge holders and that informed consent is crucial.

*Compliance with standards and ethics*: Universities (12) committed to complying with legislation, national and international standards, and protocols, including ethical guidelines concerning the use of Indigenous cultural heritage and property.

*Benefit sharing*: The policies (10) often highlighted the need for equitable sharing of benefits arising from the commercial use of Indigenous Knowledge. They called for pre-agreed terms to ensure that any commercialisation activities are fair to the Indigenous Knowledge holders.

*Consultation and negotiation*: In some policies (14), before undertaking commercial development involving Indigenous IP, there was a process of consultation and negotiation with Indigenous Knowledge Holders to agree on benefit-sharing arrangements.

*Culturally appropriate dispute resolution*: In the event of disputes regarding ICIP, one policy suggested that they should be managed respectfully and in culturally appropriate ways, often guided by specific offices or bodies within the university.

### Creator’s share of net revenue

One important aspect of research commercialisation is how the net revenue/proceeds will be distributed between the creators and the university. This was specified in the policies of 24 universities. Nine universities did not have such information in their policy, but they specified this in a ‘procedure’ document. In the case of four universities, no information was found. These universities may have such information in a document that is not publicly available, but this could also be a sign of a lack of transparency or an absence of a standardised policy in those cases. Upon reviewing this information (see Table 3), some trends can be observed.

**Table 3:** Distribution of the net revenue generated by commercialisation of research.

University	Creator/originator’s share	The rest of the net revenue
Australian Catholic University (ACU)	No information	No information
Australian National University (ANU)	Up to $50k, 100%; above $50k, 50%	Up to $50k, 0%; above $50k, 50%
Charles Darwin University (CDU)	Up to $25k, 100%; between 25 and $100k, 50%, exceeding $100k, 33.3%	Up to $25k, 0%; between 25 and $100k, 50%; exceeding $100k, 66.6%
Charles Sturt University (CSU)	No information	No information
Central Queensland University (CQU)	40%	Unit 20%, Research Division 20%, University (central) 20%
Curtin University (CU)	50%	University 50% (details unspecified)
Deakin University (DU)	33.3%	University 66.6% (half for research and half for Unit)
Edith Cowan University (ECU)	50%	Unit and Strategic Initiative Fund 50%
Federation University	50%	Unit 25% and University 25%
Flinders University	First $50k, 100%; above $50k, 40%	First $50k, 0%; above $50k, 60%
Griffith University (Griffith)	50%	Creator admin office 12.5%; Academic unit 12.5%; University 25%
James Cook University (JCU)	40%	Unit 30%, University (central) 30%
La Trobe University	50%	Unit 25%, University 25%
Macquarie University	50%	University (including unit) 50%
Murdoch University (MURD)	50%	University 50%
Monash University (Monash)	33.3%	Unit 33.3%, University 33.3%
Queensland University of Technology (QUT)	33.3%	University 66.6%
RMIT University (RMIT)	50%	Unit 25%, Research and Innovation Portfolio 25%
Southern Cross University (SCU)	33.3%	Unit 33.3%, University 33.3%
Swinburne University of Technology (SUT)	50%	University 50%
University of Adelaide	33.3%	Unit 33.3%, DVC&VP 33.3%
University of Canberra	40%	Unit 30%, University 30%
University of Melbourne	40%	Unit 40%, University 20%
University of New England (UNE)	No information	No information
University of Newcastle	First $50k 100%; between 50 and $100k, 65%; exceeding $100k, 50%	First $50k 0%; between 50 and $100k, 35%; exceeding $100k, 50%
University of Queensland (UQ)	33.3%	Unit 33.3%; University Commercialisation Company 33.3%
University of South Australia (UniSA)	40%	Unit 20%; University Venture 40%
University of Southern Queensland (USQ)	33.3%	Unit 33.33%; University 33.33%
University of Sydney (USYD)	First $25k, 100%; after that 33.3%	First $25k, 0%; after that Unit 33.3%, Vice-Chancellor’s Innovative Development Fund 33.3%
University of Tasmania (UTAS)	No information	No information
University of the Sunshine Coast (USC)	50%	Unit 25%, University 25%
University of Wollongong (UoW)	50%	50% (unspecified)
University of Western Australia (UWA)	Up to $100k, 85%; over $100k, 50%	Up to $100k, 15%; over $100k, 50%
University of New South Wales (UNSW)	33.3%	NSi (NewSouth Innovations Pty) 33.33%; University 33.33%
University of Technology Sydney (UTS)	33.3%	Unit 33.3%, University 33.3%
Victoria University (VU)	40%	Unit 30%, University 30%
Western Sydney University (WSU)	40%	Unit 30%, Research Service 30%

Notably, the distribution is not uniform; a few universities had a tiered system that adjusts the percentage based on the amount of revenue generated, while others had a flat rate regardless of the revenue amount. If we ignore the arrangement of a few universities for net revenues below $100k, the creators’ share ranges from a third to a half, with an average of approximately 42%. This suggests that a reasonable portion of the net revenue is commonly awarded to creators, acknowledging their contribution and incentivising further innovation. Universities had different approaches to the percentage of share that was not allocated to creators, however, generally, they used the rest of the net revenue to support the university’s infrastructure, including the academic units and central administration that facilitate research. Most policies (22) defined how this share is divided between different parts of the university and some others did not specify this and broadly indicated that it would go to the university. Those that specified usually gave some part of it to the academic unit to which the creator belongs (in some cases this is the school or department and in some other cases it is the faculty). Another part usually goes to the research portfolio perhaps to reinvest some of the revenue back into their research systems.

While the analysis suggests that there is no standard model for revenue sharing in Australian universities, the share of creators doesn’t vary greatly at different universities (varies between 1/3 and ½). However, different arrangements of the universities in how they divide the revenue and how they invest some of it back at the universities show a diverse landscape of policies that cater to different priorities and institutional values. This diversity could be due to various factors such as the size of the university, the focus of their research programs, and their overall strategic goals related to innovation and commercialisation. Further investigation into the impact of these policies on research output and commercialisation success would be valuable for a comprehensive understanding of their effectiveness.

## Discussion

The analysis showed that while there are similarities among IP policies of Australian universities, there are also some differences in terms of structure and content, as well as purpose, IP definition and some nuances in their approaches to IP ownership and revenue sharing. There was a variation in the structure and scope of the policies (perhaps due to different purposes discussed below). Some of the policies were high-level documents outlining things like scope, definitions, ownership, and obligation (e.g., ANU) and some others dealt with specifics such as disclosure, investigation, assessment, distribution of proceeds and so on (CQU). The other factor that contributes to differences is whether universities have a procedure document or not. Some might not have and therefore, their policy documents might serve both as a policy and a procedure.

While in the majority of universities, the policy owner was someone from the research portfolio of the Vice-Chancellor leadership team, in a few universities other officials such as university secretary was the owner. Ross [22] also found in her examination of policies that there was a diversity in responsible decision makers for activities such as identification of IP, investigation of IP viability, and finding industry partners.

A clear and inclusive definition of IP is important, especially as it is a concept that is defined in Australian federal legislation and common law (e.g. Trade Marks Act 1995, and Patents Act 1990). An overview of IP status at Australian universities in the early 1990s also suggested that without a practical and rigorous definition of what constitutes intellectual property, key questions about IP ownership could not answered [17]. Policies approached the definition of IP differently, some providing brief definitions focusing on the core concept and some providing a more nuanced definition listing different types of IP. Those that were more thorough typically reflected a comprehensive view of IP, encompassing various forms that IP can take, from tangible patents and trademarks to more intangible trade secrets and know-how.

Policies displayed a spectrum of objectives, ranging from the protection and commercialisation of IP to fostering innovation and societal benefit. This perhaps is a reflection of varying strategies and values across the Australian higher education sector, with each institution tailoring its approach to align with its unique mission and the needs of its stakeholders. Universities in Australia have different missions and stakeholders. For instance, some universities are part of the Regional Universities Network and focus on regional economy and so on, while some other universities are part of the Technology Network of Universities. However, policies collectively conveyed a strong commitment to supporting innovation, with a significant number of institutions prioritising clear rules around IP ownership and compliance. The focus on advancing and transmitting knowledge, alongside economic objectives, illustrated a dual role for universities: as custodians of knowledge and as drivers of economic growth through research commercialisation. However, there were differences in emphasis, with some universities placing greater importance on community engagement and real-world impacts than others. The presence of themes around transparent management and the protection of rights suggested a recognition of the complexities involved in managing IP and the need for principles that guide fair and effective commercialisation practices.

When IP issues emerged more widely in Australian universities in the early 1990s, the common approach was to make general claims to IP ownership and then proceed to exclude certain specified works [17]. In general, creations made by staff in the course of their employment belonged to the university, while students retained ownership of their creations unless there was an agreement to the contrary [36]. In response to some of the IP issues at the time, the National Tertiary Education Union (NTEU) developed a model IP policy [17]. Many universities’ IP policies were likely influenced by that model policy. The current review showed that there have not been major changes in IP policies with regard to the ownership of staff-created IPs. In current IP policies, all universities retain ownership of IP created by staff, particularly when such creation is related to their employment duties or involves the use of university resources. However, there is also an acknowledgment of the potential for different arrangements through agreements, reflecting a degree of flexibility in certain situations.

There has been generally a lack of advocacy for student IP rights and some experts suggest that this is an area that requires clarity in university IP policies [37]. When it comes to the ownership of IP originated from students’ work, the policies generally aimed to balance the rights of students and the encouragement of their innovation with the interests of the universities and their investment in resources and facilities. They tended to protect the university’s investments in research and education while recognising the students’ contributions. Undergraduate students usually own the IP they create unless specific conditions are met, whereas postgraduate students may be subject to more complex arrangements due to their closer involvement with research activities that are integral to the university’s mission. Conditions that result in the ownership of IP by universities include when students are employed by the university or engage in projects using significant university resources. However, as Dwyer [38] suggested, universities need to clarify when they consider their contribution significant enough to claim ownership of student-created IP.

Not all universities have specific IP rules in place for visitors, and for those that have, their approach could vary depending on the nature of the visit, the level of involvement with research activities, and the use of university resources. The situation was similar for affiliates. Some universities took a presumptive inclusion, and some took a presumptive exclusion approach with conditions that specified when the university would own the IP created by these groups. Overall, clarity of rules related to the ownership of IP created by various creator groups is critical not only to avoid legal complications but also to ensure policies are clear and easy to understand. In the early days there was not a high level of litigation concerning IP in Australian universities and Dwyer [38] maintained that this could be due to a high degree of compliance and awareness, a lack of financial resources or a lack of incentive due to the non-commercial nature of a lot of academic work. With the increasing commercialisation activities, the probability of litigation also increases, especially if policies lack clarity. Moreover, unclear university policies are a challenge for research commercialisation [39].

It is a concern that 11 policies had nothing about Indigenous cultural and intellectual property (ICIP). Those policies that had a clause or statement about ICIP, their statements suggested that there is a notable effort to approach ICIP with the highest regard for cultural sensitivity, ethical conduct, and mutual benefit. The policies express a desire not only to protect but also to actively respect and incorporate Indigenous perspectives and rights in the commercialisation of research. This is indicative of a broader recognition of the value and importance of Indigenous contributions to knowledge and the need for their active participation in the management and use of IP arising from such knowledge.

There was a bit of variation in revenue-sharing arrangements among universities with the share of creators ranging between a third and a half. It seems that compared to 25 years ago, the range has become slightly narrower as it was from 30 to 60% in the late 1990s [32]. The share of the university was usually divided between different divisions in some cases. The variation might reflect the specific context and strategic goals that each university pursues.

## Conclusion

The study underscores the importance of clear and adaptable IP policies in fostering a conducive environment for research commercialisation and innovation within academic institutions. It highlights the need for Australian universities to possibly re-evaluate their IP policies in light of the changing dynamics of research commercialisation, to ensure they remain competitive and continue to drive innovation in the global academic landscape. Based on IP policies, Australian system is predominantly a university-owned model and this might not be the best model for effective research commercialisation. For instance, there has been a call in the last few years for an overhaul of IP policies at Canadian universities for they served more as a disincentive for innovators and some experts push for a creator-owned model similar to the one adopted by the University of Waterloo [40].

The paper has some limitations. The analysis was limited to publicly available documents, and universities may outline some of the details and nuances of IP issues and commercialisation activities in other documents that are not publicly available. Future studies should look at the effectiveness of these policies and whether there is a relation between clear policies with better incentives (e.g., more revenue share for creators) and fewer barriers and more innovation and commercialisation activities at universities. Australian university policies can be compared with those of other countries with similar higher education systems but different commercialisation performances (e.g., Canada and the UK) to find out about key similarities and differences and how policies best influence innovation at universities.
